# Paraspinal Muscle Quality and Functional Outcomes Following Lumbar Fusion: A Systematic Review

**DOI:** 10.1002/msc.70299

**Published:** 2026-11-07

**Authors:** Tony Tu, Nick Kelhofer, Charlotte Cheng, Royal Shrestha, Liwan Huang, Yih‐Kuen Jan

**Affiliations:** ^1^ Carle Illinois College of Medicine University of Illinois at Urbana‐Champaign Urbana Illinois USA; ^2^ Department of Health and Kinesiology University of Illinois at Urbana‐Champaign Urbana Illinois USA

**Keywords:** erector spinae, functional outcomes, lumbar interbody fusion, multifidus, muscle atrophy

## Abstract

**Purpose:**

Lumbar interbody fusion (LIF) procedures are associated with postoperative paraspinal muscle atrophy and fatty infiltration (FI), which may influence long‐term pain and functional recovery. However, the relationship between muscle quality, both pre‐ and postoperatively, and patient‐reported outcomes remains unclear for patients undergoing lumbar fusion. This correlation was compared between minimally invasive surgery (MIS) and conventional open surgery (COS) approaches to assess differences in postoperative muscle preservation and functional outcomes.

**Methods:**

A systematic review, conducted following PRISMA guidelines and registered in PROSPERO (CRD420250650064), examined studies found in PubMed, Scopus, and Web of Science through February 2025. Studies were included if they assessed adult patients undergoing LIF with preoperative CT or MRI evaluation of the multifidus (MF) or erector spinae (ES) and reported postoperative cross‐sectional area (CSA), FI, or patient‐reported outcome measures (PROMs).

**Results:**

Twenty studies met inclusion criteria; risk of bias was assessed with ROBINS‐I for 19 non‐randomized studies and RoB 2 for one randomized trial. Seven of nine COS intervention studies found significant pre‐ to postoperative muscle damage, including all three COS‐only studies, measured by CSA and FI. Two of twelve MIS intervention studies found significant pre‐ to postoperative muscle damage, with four of six direct comparisons found smaller declines in CSA, increases in FI, or both after MIS compared to COS in at least one muscle or setting. Higher preoperative muscle quality, particularly lower FI, and smaller pre‐ to postoperative muscle changes were associated with greater PROM improvements. Across the PROM‐focused studies, four of the five reported that higher preoperative CSA or lower FI predicted better postoperative improvements in functional outcomes, with one identifying that MF FI of 61% or greater as a threshold for failing to achieve clinically meaningful ODI improvements.

**Conclusion:**

MIS techniques were associated with a decrease in pre‐ to postoperative FI compared with COS, although PROM improvement occurred across both approaches. While preoperative paraspinal muscle quality and size decreases are generally smaller with better functional outcomes following postoperative LIF, this relationship is not consistently observed across all studies or surgical techniques. These findings suggest that the current imaging‐based biomarkers may not fully capture the multifactorial nature of functional recovery and underscore the need for further research into additional predictors of muscle recovery.

**Abbreviations**
COS: Conventional open surgery
CSA: Cross‐sectional area
CT: Computed tomography
ES: Erector spinae
FI: Fatty infiltration
LBP: Lower back pain
LIF: Lumbar interbody fusions
LLIF: Lateral lumbar interbody fusion
LM: Lumbar muscularity
MF: Multifidus
MIS: Minimally invasive surgery
MRI: Magnetic resonance imaging
ODI: Oswestry disability index
OLIF: Oblique lateral interbody fusion
PCS: Physical component summary
PLIF: Posterior lumbar interbody fusion
PRISMA: Preferred Reporting Items for Systematic Reviews and Meta‐Analyses
PROMs: Patient‐reported outcome measures
TLIF: Transforaminal lumbar interbody fusion
VAS: Visual analog pain scale

## 1 Introduction

Between 1998 and 2008, the annual number of spinal fusion hospital discharges increased from 174,223 to 413,171, increasing by 137% in ten years alone (Rajaee et al. 2012). Although these procedures stabilise the spine, they can also impact the integrity of the paraspinal muscles (Hyun et al. 2007). This is especially relevant in lumbar interbody fusion (LIF) procedures, which include posterior (PLIF), transforaminal (TLIF), oblique (OLIF), and lateral (LLIF) approaches. While PLIF is commonly employed because it provides a wider surgical view, TLIF and LLIF utilises a unilateral approach sparing the contralateral musculature via the intervertebral foramen lateral aspect of the vertebral body, respectively (Mobbs et al. 2015; Yoshihara and Yoneoka 2015). Either approach can be performed using conventional open surgery (COS) or minimally invasive surgery (MIS) techniques. MIS reduces muscle disruption in return for higher cost and a steeper learning curve (Zileli et al. 2024). Although MIS and COS procedures aim to preserve muscle integrity, the extent to which paraspinal muscle size and quality are associated with patient factors and patient‐reported outcome measures (PROMs) remains poorly understood.

When studying surgical outcomes, muscle quality is typically assessed using biomarkers like cross‐sectional area (CSA) and fatty infiltration (FI), allowing for comparison between different surgical methods (Fu et al. 2020; Ghiasi et al. 2016; Peng et al. 2020). A decrease in CSA or volume and an increase in FI all indicate a higher degree of muscle damage. These biomarkers are measured using either computed tomography (CT) or magnetic resonance imaging (MRI). PROMs, most commonly Oswestry disability index (ODI) and visual analog pain scale (VAS), are used to evaluate functional impairment and pain level, respectively. Higher degrees of FI and paraspinal atrophy are generally associated with worse scores on these tests.

Despite these different imaging techniques and outcome measures, there remains limited clarity on how changes in muscle quality and size, both pre‐ and postoperatively, translate into functional recovery. This has prompted researchers to investigate the correlation between pre‐ and postoperative muscle quality and size, surgical success, and complications following LIF (Han et al. 2023). In addition, this has also fuelled interest in identifying predictors of functional outcomes due to the clinical variability in postoperative outcomes of spinal surgeries (Marchand et al. 2022). While previous reviews have explored changes in muscle quality and size, after surgery or examined the association between muscle health and patient outcomes, few have evaluated how these elements interact across different surgical techniques (Han et al. 2023). Our comprehensive analysis directly compares pre‐ and postoperative muscle biomarkers with PROMs across MIS and COS approaches. In addition, while some reviews have investigated the relationship between preoperative muscle quality and size, and surgical complications, as well as how these complications affect PROMs, there remains a gap in understanding on how preoperative muscle quality and size directly influence PROMs (Gengyu et al. 2022). This study aims to (1) evaluate changes in multifidus (MF) and erector spinae (ES)'s quality as measured by FI, and muscle size as measured by CSA, before and after PLIF, TLIF, OLIF, and LLIF procedures; (2) correlate these findings with expected and actual PROMs; and (3) assess how MIS and COS LIF influence paraspinal muscle size, quality, and healing. Evaluating these relationships may help us better understand the mechanisms of postoperative healing and inform clinical care and rehabilitation strategies.

## 2 Methods

### 2.1 Registration and Protocol

The Preferred Reporting Items for Systematic Reviews and Meta‐Analyses (PRISMA) statement was used to structure this systematic review (Moher et al. 2009). The review protocol was registered with PROSPERO, an international database for systematic reviews, on February 12th, 2025, under the registration number: CRD420250650064. The protocol was followed except that a single randomized trial was assessed with RoB 2 rather than ROBINS‐I, and the full protocol details can be accessed on the PROSPERO database (Tu et al. 2025).

### 2.2 Databases and Search Strategy

To retrieve articles, we searched PubMed, Scopus, and Web of Science for literature published through February 27, 2025 using the following search terms (‘back muscle’ OR ‘paraspinal muscle’ OR ‘multifidus’ OR ‘paravertebral’ OR ‘erector spinae’) AND (‘Lumbar fusion’ OR ‘lumbar interbody fusion’ OR ‘TLIF’ OR ‘PLIF’ OR ‘LLIF’ OR ‘ALIF’). The full search strategy for each database is provided in Table S1. Reference lists of all included articles were also searched for relevant articles. Two reviewers (TT, NK) independently assessed titles and abstracts to determine eligibility for further review and then conducted a full text evaluation based on inclusion and exclusion criteria. All discrepancies were resolved through discussion and evaluation by a third reviewer (CC). The screening process was managed with Zotero.

### 2.3 Inclusion and Exclusion Criteria

Studies included in this review met all of the following criteria: (1) subjects are adults, age ≥ 18, who received LIF, regardless of underlying indication; (2) preoperative assessment of paraspinal muscle, defined as MF and/or (ES), using CT or MRI; (3) postoperative assessment of paraspinal muscle using CT or MRI OR postoperative assessment of surgical outcome using PROMs; and (4) published in English. Studies were excluded from the review if they met one or more of the following criteria: (1) did not report preoperative assessment of paraspinal muscles; (2) used means other than CT or MRI; (3) did not analyze the MF or ES; and (4) or only evaluated experimental LIF techniques. Eligible study designs included randomized controlled trials, cohort studies, case–control studies, and case series, while abstracts and individual case reports were excluded.

### 2.4 Data Extraction

A data extraction table was used to collect the following methodological and outcome variables from each study: author(s)/publication year, sample size, participants' age, participants' sex, study design, condition(s) for surgery vertebral level treated, surgical technique, muscle(s) evaluated, imaging modality, PROMs reported, muscle metrics, follow‐ups, and association results. The tables were extracted by TT and CC independently for Table 1 and reviewed by NK, NK for Table 2 and reviewed by TT, and RS for Table 3 and reviewed by NK. All tables were then reviewed by TT and YJ.

**TABLE 1:** Study characteristics and postoperative changes in paraspinal muscle morphology following lumbar interbody fusion, including comparisons between surgical approaches.

Author/year	Study design	Mean age	*n*, (% male)	Condition(s) for surgery	Intervention(s)	Muscle(s) assessed (level(s))	Imaging modality	PROMs reported	Muscle metrics	Follow‐ups	Results
Pang et al. (2022)	Retrospective cohort	61.0 ± 8.9	33 (39%)	Lumbar spinal stenosis	MIS‐TLIF	MF (L3/4 and L5/S1)	CT	ODI, VAS	CSA, FI	Imaging: 1 year, 2 years, 3 years Clinical: 1 week, 3 months, 12 months, > 3 years	Neither functional CSA nor FI significantly changed following the operation (*p* > 0.05).
Moser et al. (2023)	Retrospective cohort	64.3 ± 10.6	67 (45%)	Degenerative conditions	SA‐LLIF	Psoas, ES and MF (L2/3 to L4/5)	MRI	N/A	CSA, FI	3–18 months	At the L4/5 level, there was a significant increase in psoas total CSA (+4.8% ± 12.4%; *p* = 0.013). At the L3/4 and L4/5 levels, there was a significant increase in paraspinal muscle FI (L3/4: +3.1% ± 6.5%; *p* = 0.002; L4/5: +3.0% ± 7.0%; *p* = 0.002).
Kim and Wu (2024)	Retrospective cohort	MIS‐TLIF: 68.67 (41−86) Endo‐TLIF: 64.76 (39−82)	116 (24%)	Spondylolisthesis and spinal stenosis with segmental instability	Single level Endo‐TLIF and MIS‐TLIF	Psoas and paraspinal muscle (ipsilateral upper mid pedicle for the level of TLIF)	MRI	ODI, VAS	CSA, FI	MIS TLIF: 24–70 months Endo TLIF: 12–31 months	Patients undergoing MIS TLIF had no change in psoas CSA (right: −22.28, *p* = 0.295; left: −15.52, *p* = 0.462) but had significant decrease in bilateral paraspinal muscles (right: −89.88, *p* = 0.003; left: −132.74, < 0.001) postoperatively.
Fu et al. (2020)	Prospective cohort	COS: 59.2 ± 8.6, MIS 60.2 ± 10.0	46 (41%)	Segmental instability, spondylolisthesis, and disc degeneration disease with herniation and/or spinal stenosis	TLIF	Paraspinal muscle and dural sac (L2‐3 to L5‐S1)	MRI	ODI, JOA	CSA, FI	1 year	Patients in the COS group experienced a greater increase in FI (27.37% ± 21.37%) compared to the MIS group (14.13% ± 19.19%) in the ES (*p* = 0.044). No significant differences in FI changes were found between the COS and MIS groups in the MF at the operated and cranial adjacent levels.
Putzier et al. (2016)	Prospective non‐blinded clinical trial	PLIF: 64.0 (44–86), TLIF: 61.7 (39–84)	47 (43%)	Single‐level segment degeneration	TLIF, PLIF	Posterior paraspinal muscles (L3/L4 to L5/S1)	CT	ODI, VAS	Volume, FI	1 week, 1 year	MF residual volume at 12 months (relative to 1 week postoperatively) was 96.6% in patients in the MIS TLIF group versus 45.8% in patients in the COS PLIF group (*p* < 0.001), and MF fat content rose to 19.3% versus 58.6% (*p* < 0.001). Longissimus volume loss was similar between groups; longissimus fat content rose about 3% more after COS PLIF (*p* = 0.032).
Cho et al. (2020)	Retrospective cohort	59.6 ± 12.1	40 (20%)	Degenerative lumbar spinal stenosis	PLIF	MF + ES (L3 lower endplate)	MRI, CT	N/A	CSA, volume, FI	1 year	Patients exhibited a significant reduction in MF volume bilaterally on both MRI (right: 41.6–24.3 cm^3^, *p* = 0.003; left: 40.8–23.5 cm^3^, *p* < 0.001) and CT (right: 42.2–34.1 cm^3^, *p* = 0.005; left: 41.9–32.3 cm^3^, *p* < 0.001). In the MRI group, no significant change in ES volume was observed. In the CT group, a significant decrease was noted in the right‐sided ES muscle (130.8–120.5 cm^3^, *p* < 0.001). No significant changes were observed in psoas muscle volume in either group.
Kolcun et al. (2025)	Retrospective cohort	67.1 ± 5.7	10 (40%)	Spinal deformity	MIS‐LLIF	MF + ES	MRI	N/A	CSA, volume, FI	2 years	No significant reduction in ES, MF, or psoas percent fatty area (all *p* > 0.05).
He et al. (2020)	Retrospective cohort	OLIF: 59.8 ± 13.7 Combined OLIF: 61.0 ± 9.3 years	73 (29%)	Spondylolisthesis	OLIF and combined OLIF	MF + ES (L3‐L5)	CT	VAS	CSA, FI	2 years	There were no significant changes in MF CSA (*p* = 0.072), MF FI (*p* = 0.721), ES CSA (*p* = 0.533), or ES FI (*p* = 0.578).
Kameyama et al. (2023)	Retrospective cohort	PLIF: 71.4 ± 7.2 MIS‐LLIF: 70.8 ± 8.9	62 (0%)	Spondylolisthesis, discitis	PLIF and MIS‐LLIF	MF + ES (N/A)	CT	N/A	CSA, volume, FI	6 months, 1 year	No significant difference was observed in CSA changes across groups for any paraspinal muscle (*p* > 0.05). Patients in the open PLIF group had decreased MF density (−5.8 ± 1.2 hounsfield units, *p* < 0.05) and an increased psoas density (+3.4 ± 0.9 hounsfield units, *p* < 0.05) at 1 year. Patients in the MIS‐LLIF group had no decrease in MF density (*p* > 0.05) and increased psoas density (+4.1 ± 1.0 hounsfield units, *p* < 0.05).
Xu et al. (2024)	Retrospective cohort	57.06 ± 10.66	149 (47.67%)	Degenerative lumbar spinal stenosis	PLIF	MF + ES (L2‐S1)	MRI	NA	Relative CSA, functional CSA, total CSA, FI	Mean: 25.71 ± 8.35 months	For the levels L5‐S1, there was decreased CSA and increased FI in the MF (both CSA and FI *p* < 0.001) and ES (both CSA and FI *p* < 0.001).
Min et al. (2009)	Prospective controlled cohort	57 (30–77)	48 (35.41%)		PLIF and MI‐TLIF	Paraspinal muscles (L1‐L5)	MRI	NA	CSA, volume, FI	1 year	Neither patients in the COS‐TLIF nor the MI‐TLIF group had change in paraspinal muscle FI (*p* = 0.124) or CSA (*p* = 0.175).
Li et al. (2022)	Prospective cohort	63.63 ± 9.51	30 (46.7%)	Lumbar spinal stenosis	PLIF	Paraspinal muscles (L4‐L5)	MRI	VAS, ODI	CSA, volume, FI	1, 3, 6 months, and 1 year	There was a significant difference in FI of the upper segment of the ES before and after surgery (*Z* = −2.00, *p* < 0.05).
Waschke et al. (2014)	Prospective cohort	60.5 (33–80)	30 (40.6%)	Spinal stenosis	PLIF (2‐ or 3‐segment)	MF + ES (L3‐S1)	CT, EMG	SF36, VAS	FI, volume	3, 6, 12 months	Patients who underwent PLIF had a significant decrease in paraspinal muscle tissue percent (67.8%–60.4%, *p* < 0.001) and an increase in FI percentage (29.5%–39.7%, *p* < 0.001) at 12 months post‐operative.
Fan et al. (2010)	Prospective cohort	MIS ‐ 53.3 ± 9.9 CO ‐ 53.0 ± 11.6	59 (44.1%)	Spinal stenosis, huge lumbar disc herniation, or low‐grade spondylolisthesis	Single‐level conventional PLIF or MIS‐PLIF	MF (L3‐S1)	MRI,	ODI, VAS	CSA	Mean 14 months	At the level fused, magnitude change in MF CSA at 12 months post‐operative was different between patients treated with TLIF (relative CSA = −36.8 ± 12.3) and MIS‐PLIF group (relative CSA = −12.2 ± 4.5) (*p* < 0.01). At the adjacent level, magnitude change in MF CSA at 12 months post‐operative was different between patients treated with TLIF (relative CSA = −29.3 ± 13.3) and MIS‐PLIF group (relative CSA = −8.5 ± 3.9) (*p* < 0.01).
K. Chen et al. (2021)	Retrospective cohort	66	81 (42%)	Low back pain or claudication	TLIF, MIS‐TLIF (2‐ or 3‐segment)	MF (L3‐S1)	MRI	VAS, ODI	CSA, volume, FI	3, 6, 12 months	At 12 months post‐operative, relative CSA change was higher for patients who received 3‐segment TLIF group (= −361.1 ± 83.6) compared to 3‐segment MIS‐TLIF (relative CSA = −267 ± 82) (*p* < 0.01). Relative CSA change was not significantly different between approaches for 2‐segment fusion.

*Note:* Unless otherwise stated, changes are postoperative minus preoperative values; negative CSA or volume changes indicate muscle loss.

Abbreviations: COS, conventional open surgery; CSA, cross‐sectional area; CT, computed tomography; EMG, electromyography; Endo‐TLIF, endoscopic transforaminal lumbar interbody fusion; ES, erector spinae; FI, fatty infiltration; JOA, Japanese Orthopaedic Association score; LLIF, lateral lumbar interbody fusion; MF, multifidus; MIS, minimally invasive surgery; MRI, magnetic resonance imaging; ODI, Oswestry Disability Index; OLIF, oblique lateral interbody fusion; PLIF, posterior lumbar interbody fusion; SA‐LLIF, standalone lateral lumbar interbody fusion; SF‐36, 36‐Item Short Form Health Survey; TLIF, transforaminal lumbar interbody fusion; VAS, visual analog scale.

**TABLE 2:** Study characteristics and associations between paraspinal muscle morphology and postoperative patient‐reported outcomes.

Author/Year	Design	Mean age	n, (% male)	Condition(s) for surgery	Intervention(s)	Muscle(s) assessed (Level(s))	Imaging modality	PROMs reported	Muscle metrics	Follow‐ups	Results
Waschke et al. (2014)	Prospective cohort	60.5 (33–80)	30 (40.6%)	Spinal stenosis	PLIF (2‐ or 3‐segment)	MF + ES (L3‐S1)	CT	SF36, VAS	FI, volume	3, 6, 12 months	Mean postoperative paraspinal muscle volume was associated with SF‐36 physical component scores (*r* = 0.169; *p* = 0.02), mental component scores (*r* = 0.214; *p* = 0.003), and pain scores (*r* = 0.382; *p* < 0.001).
Zavras et al. (2024)	Retrospective cohort	57.77 ± 10.12	74 (52.7%)	Isthmic spondylolisthesis, degenerative spondylolisthesis, severe degenerative disc disease, or foraminal stenosis	LLIF (1‐, 2‐, or 3‐segment)	MF (L3‐S1)	MRI	ODI, VAS, SF12, rand 12	Goutallier grade, kader class	6 weeks, 1 year	At 6 weeks, greater preoperative MF atrophy was associated with worse SF‐12 scores (kader: *R* = 0.295, *p* = 0.049; goutallier: *R* = 0.354, *p* = 0.017) and VR‐12 scores (goutallier: *R* = 0.335, *p* = 0.025). At 1 year, associations were reported with ODI (kader: *R* = 0.279, *p* = 0.028) and SF‐12 (goutallier: *R* = 0.294, *p* = 0.047).
You et al. (2023)	Retrospective cohort	67.17 ± 7.37	206 (33.98%)	Spinal stenosis or low‐grade spondylolisthesis	PLIF or MIS‐TLIF	Paraspinal muscles (L3 lower endplate)	MRI, CT	VAS, ODI	Lumbar muscularity (CSA), FI	1 year	High lumbar muscularity was associated with lower 1‐year VAS than low muscularity (1.2 ± 1.2 vs. 1.8 ± 1.4; *p* = 0.004). The high‐muscularity group had greater 1‐year ODI improvement than the medium group (30.1 ± 11.9 vs. 24.2 ± 12.1 points; *p* = 0.018). Lower FI was associated with greater ODI improvement at 3 months (*p* < 0.001) and 6 months (*p* = 0.003).
Amorim‐Barbosa et al. (2023)	Retrospective cohort	52 (22–76)	75 (32%)	Degenerative disc disease, lumbar spinal stenosis, spondylolisthesis, and adult spinal deformity	PLIF and TLIF	MF and posterior paraspinal muscles (L4/5 and L5/S1)	MRI	ODI, VAS	FI	2 years	Postoperative ODI and back and leg pain VAS scores did not differ significantly across FI groups. The most severe FI group showed less improvement in leg pain than the lowest FI group (81% vs. 97%; *p* = 0.04).
Schönnagel et al. (2024)	Retrospective analysis of prospectively enrolled patients	67.9 (62–72.8)	133 (28.6%)	Degenerative lumbar spondylolisthesis	LIF	ES and MF (L4 upper plate)	MRI	ODI	CSA, FIR	2 years	Greater preoperative FI was associated with higher odds of failing to achieve clinically meaningful ODI improvement: MF OR = 1.05 (95% CI 1.02–1.08; *p* < 0.004) and ES OR = 1.04 (95% CI 1.01–1.09; *p* < 0.025).
J. Chen et al. (2022)	Retrospective analysis of prospectively enrolled patients	51.4 ± 7.3	58 (51.72%)	Lumbar spondylolisthesis	LLIF	MF (L4‐L5)	MRI	VAS, ODI	CSA, FIR	1 year	Higher preoperative MF CSA ratio was associated with lower preoperative and postoperative VAS and ODI scores (*p* < 0.001).

*Note:* Muscle assessments were preoperative except in Waschke et al. (2014), which examined associations with postoperative muscle volume.

Abbreviations: CSA, cross‐sectional area; CT, computed tomography; ES, erector spinae; FI, fatty infiltration; FIR, fat infiltration rate; LLIF, lateral lumbar interbody fusion; MF, multifidus; MIS, minimally invasive surgery; MRI, magnetic resonance imaging; ODI, Oswestry Disability Index; OR, odds ratio; PLIF, posterior lumbar interbody fusion; PROMs, patient‐reported outcome measures; SF‐12, 12‐Item Short Form Health Survey; SF‐36, 36‐Item Short Form Health Survey; TLIF, transforaminal lumbar interbody fusion; VAS, visual analog scale; VR‐12, Veterans RAND 12‐Item Health Survey.

**TABLE 3:** Study characteristics and associations between preoperative‐to‐postoperative changes in multifidus cross‐sectional area and postoperative pain and disability.

Author/Year	Design	Mean age	*n* = (%male)	Condition(s)	Intervention(s)	Muscle(s) assessed (Level(s))	Imaging modality	PROMs reported	Muscle metrics	Follow‐ups	Results
K. Chen et al. (2021)	Retrospective cohort	66	81 (42%)	Low back pain or claudication	TLIF, MIS‐TLIF (2‐ or 3‐segment)	MF (L3‐S1)	MRI	VAS, ODI	CSA, volume, FI	3, 6, 12 months	MF CSA change was associated with ODI at 12 months after three‐level TLIF (*r* = 0.36; *p* < 0.03), but not after two‐level TLIF (*r* = 0.11; *p* = 0.52). Associations with VAS were not significant after either three‐level (*r* = 0.12; *p* = 0.45) or two‐level TLIF (*r* = 0.16; *p* = 0.34).
Fan et al. (2010)	Prospective cohort	MIS—53.3 ± 9.9 COS—53.0 ± 11.6	59 (44.1%)	Spinal stenosis, huge lumbar disc herniation, or low‐grade spondylolisthesis	Single‐level conventional PLIF or MIS‐PLIF	MF (L3‐S1)	MRI	ODI, VAS	CSA, at multiple levels	Mean 14 months	Preoperative‐to‐final‐follow‐up MF CSA change was associated with ODI (*r* = −0.718; *p* < 0.001) and VAS (*r* = −0.798; *p* < 0.001).

*Note:* Correlation signs are retained as reported and should be interpreted according to each study's definition of CSA change.

Abbreviations: COS, conventional open surgery; CSA, cross‐sectional area; FI, fatty infiltration; LBP, low back pain; MF, multifidus; MIS, minimally invasive surgery; MRI, magnetic resonance imaging; ODI, Oswestry Disability Index; PLIF, posterior lumbar interbody fusion; PROMs, patient‐reported outcome measures; TLIF, transforaminal lumbar interbody fusion; VAS, visual analog scale.

### 2.5 Quality Assessment

Quality of included studies was assessed independently by two reviewers, CC and RS, using the ROBINS‐I (J. A. Sterne et al. 2016) and RoB 2 (J. A. C. Sterne et al. 2019) tool. The ROBINS‐I tool was selected because the included studies were non‐randomized and observational in design, making it an appropriate choice for evaluating risk of bias in intervention studies. The RoB 2 tool was used for a single randomized trial. CC and RS assessed the quality of each study and identified potential sources of bias according to guidance from Sterne et al. (2016). Any discrepancy in judgement was resolved through consensus discussion, with adjudication by a third reviewer, TT if needed. Quality assessment organisation and figure generation were performed using the robvis tool (McGuinness and Higgins 2021).

### 2.6 Data Synthesis

Because of substantial differences in study design and techniques among the included studies, meta‐analysis was not feasible. Instead, a narrative synthesis was performed, summarising findings across studies while considering variations in study design, interventions, and outcome measures. The results were organised thematically to highlight similarities and differences in study outcomes.

## 3 Results

### 3.1 Study Characteristics and Risk of Bias

Our PubMed, Scopus, and Web of Science searches yielded 982 studies. 490 duplicate studies were removed and 492 studies were screened for exclusion and inclusion criteria. The screening resulted in 81 studies being eligible for a full text screen. After the full text screening, twenty unique studies were included in this systematic review (Figure 1).

**FIGURE 1:**
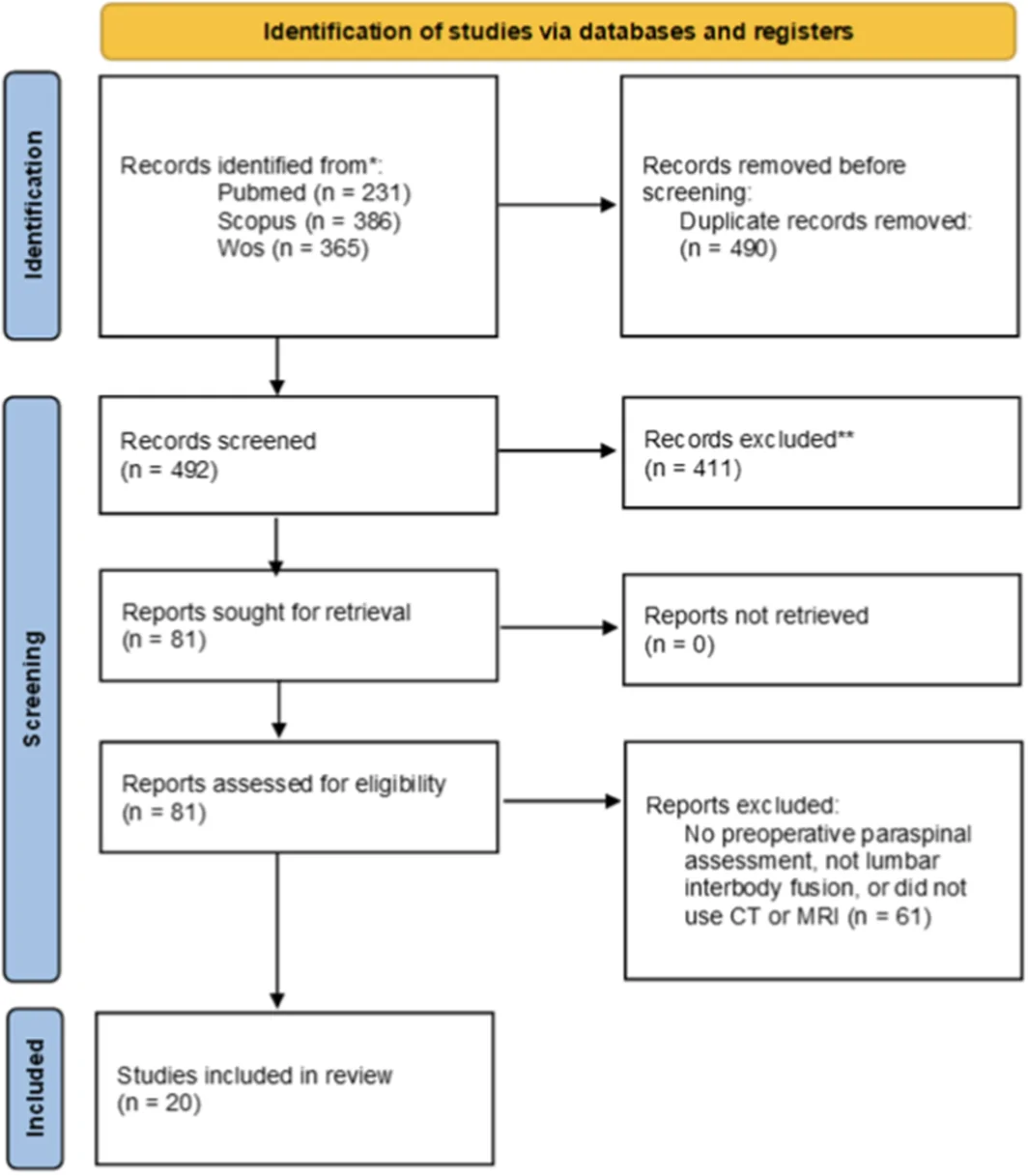
PRISMA flow chart of the study selection process.

The included studies investigated LIF using both MIS and COS of the following surgical methods: LLIF, PLIF, and TLIF. The studies included were prospective cohorts, retrospective cohorts, prospective clinical trials, and retrospective analysis of prospectively enrolled patients. Conditions included in the studies reviewed include low back claudication, thoracolumbar fractures, osteosarcopenia, degenerative disc disease, adult spinal deformity, lumbar spondylolisthesis, and lumbar spinal stenosis.

The robvis ROBINS‐I Cochrane Reviews risk of bias assessment tool was used to assess the nineteen non‐randomized studies, and the robvis RoB 2 risk of bias assessment tool was used to assess a single randomized trial. Out of the non‐randomized studies, we found five studies with a low risk of bias, eleven studies with a moderate risk of bias, and three studies with a serious risk of bias (Figure 2a,c). The randomized trial (Putzier et al. 2016) was assessed for some concerns of risk. There are zero studies in this review with a critical risk of bias (Figure 2c,d).

**FIGURE 2:**
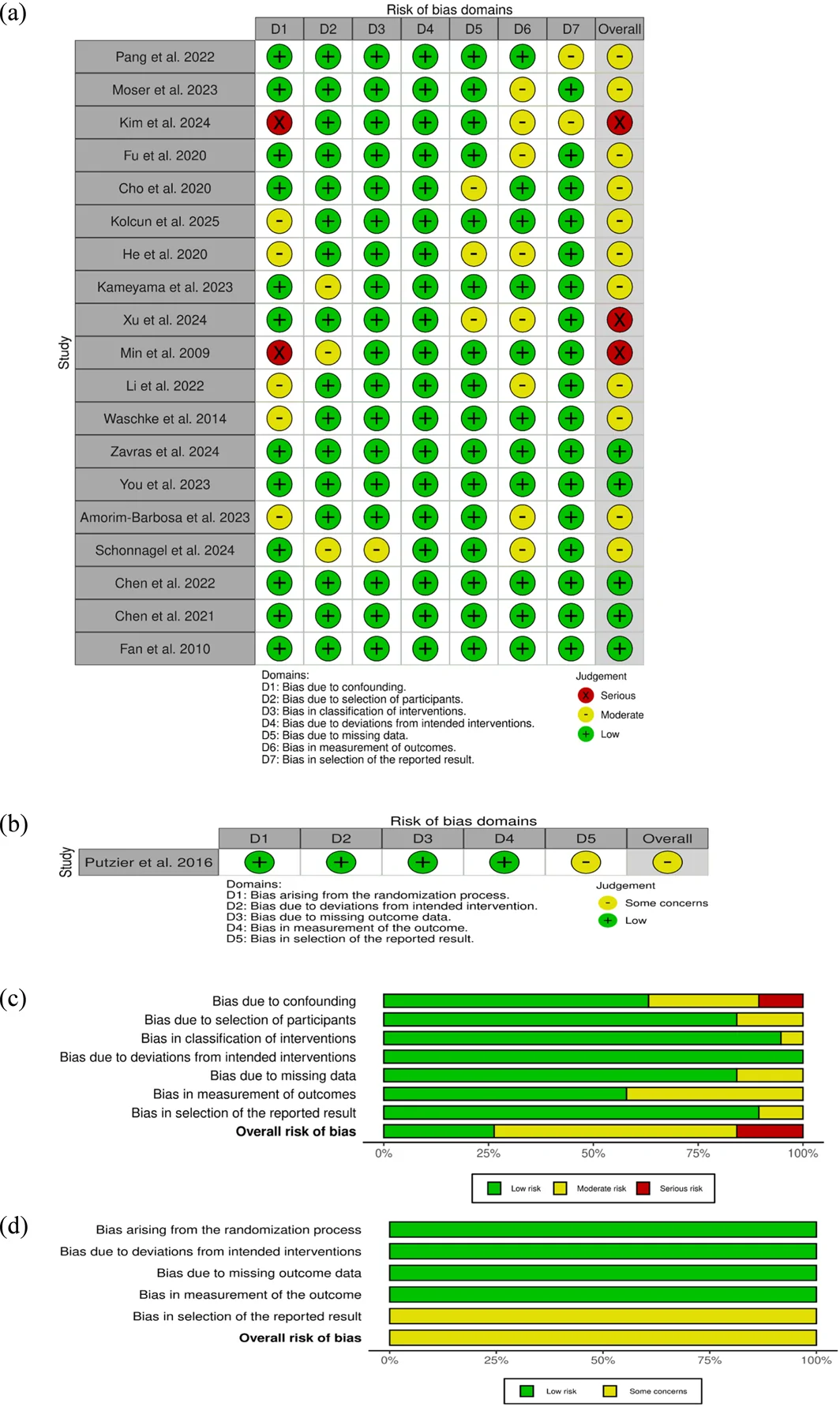
(a) Robvis ROBINS‐I cochrane reviews risk of bias for the included non‐randomized studies. (b) Robvis RoB 2 cochrane reviews risk of bias for the included randomized controlled trials. (c) Robvis ROBINS‐I and (d) RoB 2 cochrane reviews risk of bias summary.

### 3.2 Minimally Invasive Procedures Correlate With Lower Preoperative to Postoperative Changes in Muscle Cross‐Sectional Area and Fatty Infiltration

We identified fifteen studies reporting the changes in CSA and FI after MIS or COS surgeries. Of these, three studies compared the CSA and FI between the pre‐ and post‐operative images for COS interventions only, six studies evaluated MIS only, and six studies compared the change in CSA and FI between MIS and COS (Table 1).

Of the three studies discussing COS only, all three studies found a statistically significant pre‐ to postoperative decrease in muscle CSA, increase in muscle FI, or both. Cho et al. (2020) found a decrease in pre‐ to postoperative muscle volume in MF (Right: 42.2–34.1 cm^3^, *p* = 0.005) (Cho et al. 2020). There was also a significant decrease in ES volume (Right: 130.8–120.5 cm^3^, *p* < 0.001). Xu et al. (2024) found a decreased total CSA and increased FI in MF for the L5‐S1 lumbar levels (CSA 0.53 ± 0.18 to 0.42 ± 0.13, FI 0.31 ± 0.10 to 0.71 ± 0.19, *p* < 0.001 for both) (Xu et al. 2024). Similarly, the ES muscle was found to also have a decreased total CSA and increased FI for the L5‐S1 lumbar levels (CSA 0.43 ± 0.2 to 0.19 ± 0.11, FI 0.38 ± 0.15 to 0.52 ± 0.19, *p* < 0.001 for both). Waschke et al. (2014) found a significant decrease in percent muscle volume (67.8%–60.4%, *p* < 0.001) and an increase in % FI (29.5%–39.7%, *p* < 0.001) (Waschke et al. 2014).

In total, six papers discuss only MIS approaches. Of those, four studies found that there was no statistically significant difference in CSA and FI, with results varying by approach. Pang et al. (2022) found that there was no statistically significant change in functional CSA, which is defined as total muscle CSA minus fat CSA, of the MF between the pre‐operation and all subsequent follow‐ups (*p* > 0.05) (Pang et al. 2022). They also found that FI of the MF did not change significantly at subsequent follow‐ups (*p* > 0.05). Similarly, Kolcun et al. found that there was no significant increase in ES or MF percent fat area (Right ES: −3.8, *p* = 0.678, Right MF: 6.4 *p* = 0.496, respectively) (Kolcun et al. 2025). Likewise, Li et al. (2022) found that there was no significant difference in ES CSA and FI (*p* > 0.05) (Li et al. 2022). He et al. (2020) found that standalone OLIF was not followed by significant changes in MF CSA (CSA 8.59 ± 1.76 cm^2^ to 9.39 ± 1.74 cm^2^, *p* = 0.072) and ES CSA (13.32 ± 1.59 cm^2^ to 13.55 ± 1.31 cm^2^, *p* = 0.533). This paper also found no significant change in MF FI (FI 15.91 ± 5.30% to 14.38 ± 3.21%, *p* = 0.721) and ES FI (FI 11.63 ± 3.05% to 11.22 ± 3.12%, *p* = 0.578) (He et al. 2020). Kim and Wu (2024) found that there is no significant change in left and right psoas CSA (right: −22.28, *p* = 0.295; left: −15.52, *p* = 0.462) but found a significant decrease in bilateral paraspinal muscles (right: −89.88, *p* = 0.003; left: −132.74, < 0.001) (Kim and Wu 2024). However, Moser et al. (2023) found a significant increase in total CSA (+4.8 ± 12.4%; *p* = 0.013) and FI (L3/4: +3.1 ± 6.5%; *p* = 0.002) in the paraspinal muscles (Moser et al. 2023).

Of the fifteen studies, six studies discussed and compared both MIS and COS. Four of the studies found that MIS surgeries showed smaller declines in CSA or smaller increases in FI in at least one muscle group, whereas the other two studies found no significant differences in imaging biomarkers between MIS and COS. Fu et al. (2020) reported that there was an increase in FI in COS compared with MIS in the ES (27.37 ± 21.37%, *p* = 0.044) (Fu et al. 2020). Putzier et al. (2016) found that MF volume was largely preserved after MIS TLIF but fell by more than half after COS PLIF (residual volume at 12 months relative to 1 week postoperatively, 96.6% vs. 45.8%; *p* < 0.001), whereas longissimus volume loss was similar between groups (Putzier et al. 2016). MF FI at 12 months was also lower after MIS than COS (median 19.3% vs. 58.6%; *p* < 0.001). Fan et al. also found that there was a significant difference between a decrease in muscle CSA when comparing COS and MIS procedures in the level of the fusion (COS: CSA = −36.8 ± 12.3 MIS: −12.2 ± 4.5, *p* < 0.01) (Fan et al. 2010). Similar findings were seen in the adjacent levels (COS: CSA = −29.3 ± 13.3 MIS: −8.5 ± 3.9, *p* < 0.05). Chen et al. (2021) found a significantly larger decrease in MF CSA at 12 months post‐operative in COS when compared to MIS for 3‐segment TLIF (−361.1 ± 83.6 vs. −267.0 ± 82.0; *p* < 0.01), but also found no significant decrease in MF CSA at 12 months post‐operative in COS when compared to MIS for 2‐segment TLIF (−144.0 ± 63.9 vs. −127.1 ± 49.6; *p* = 0.45), although the trend is still that COS causes greater reduction is MF CSA (K. Chen et al. 2021). However, Kameyama et al. found that both COS and MIS did not have significant changes in CSA (Kameyama et al. 2023). Similarly, Min et al. found no significant difference in FI or CSA between the two surgical approaches (Min et al. 2009).

Overall, all three COS‐only studies reported decreased paraspinal muscle size, increased FI, or both after surgery, and four of the six direct comparisons found smaller changes after MIS than after COS, although some of these differences were limited to specific muscles. Of the six MIS‐only studies, four found no significant change, whereas two reported a significant decrease in CSA or increase in FI, depending on the technique. Because the studies used different muscles, metrics, and imaging modalities, the available findings do not establish the magnitude of differences between approaches.

### 3.3 Paraspinal Cross Sectional Area and Quality Correlates With Patient‐Reported Outcome Measures

Six studies evaluating preoperative muscle morphology reported variable associations with postoperative pain and disability (Table 2). The analyses differed in whether they compared final postoperative scores, changes from baseline, or achievement of clinically meaningful improvement. Across the studies, associations between paraspinal muscle morphology and postoperative pain or disability varied by muscle measure, outcome, and assessment time point (Table 2).

You et al. (2023) reported lower 1‐year VAS scores in patients with high versus low lumbar muscularity, defined by the ratio of MF CSA to CSA of the L3 lower endplate (1.2 ± 1.2 vs. 1.8 ± 1.4; *p* = 0.004) (You et al. 2023). The group with higher initial muscularity also showed greater ODI improvement than the medium‐muscularity group (30.1 ± 11.9 vs. 24.2 ± 12.1 points; *p* = 0.018). Similarly, patients in the lowest fatty‐infiltration category showed greater ODI improvement at 3 and 6 months than patients in higher categories. This cohort included different surgical approaches, but associations were not stratified by approach. For patients undergoing LLIF, Chen et al. (2022) found that a higher MF CSA ratio, defined by the ratio of muscle to vertebral size, was associated with lower VAS and ODI scores, both preoperatively and postoperatively (both *p* < 0.001) (J. Chen et al. 2022). Similarly, less FI was associated with lower VAS scores both preoperatively and postoperatively (both *p* < 0.001). They did not report an association between change in CSA and outcome measures. Schönnagel et al. (2024) reported associations between failure to achieve clinically meaningful ODI improvement and greater preoperative multifidus (*p* < 0.001) and erector spinae (*p* = 0.01) fatty infiltration (Schönnagel et al. 2024). Specifically, patients with a MF FI ≥ 61% were 5.1 times more likely to not have a significant improvement in ODI score (95% CI: 1.88–15.30, *p* = 0.002). Zavras et al. (2024) also reported associations between poorer preoperative multifidus quality and selected postoperative PROMs following LLIF, with findings varying by grading method, outcome, and follow‐up time (Zavras et al. 2024). Notably, MF atrophy was associated with worse ODI (*p* = 0.028) and SF12 (*p* = 0.047) at one year after surgery.

The association was not uniform across studies. Amorim‐Barbosa et al. (2023) reported no statistically significant differences in postoperative ODI or back and leg pain VAS scores across fatty‐infiltration groups, although the most severe fatty‐infiltration group showed less improvement in leg pain (Amorim‐Barbosa et al. 2023).

Finally, Waschke et al. (2014) examined postoperative muscle morphology rather than pre‐operative predictors of recovery. Following open PLIF, VAS scores decreased from 7.8 preoperatively to 4.7 at 1 year despite increased FI and muscle atrophy (Waschke et al. 2014). Postoperative paraspinal muscle volume was associated with SF‐36 physical (*p* = 0.02), mental (*p* = 0.003), and pain‐related (*p* < 0.001) outcomes, indicating that clinical improvement and morphological deterioration could co‐exist.

Overall, greater preoperative FI and lower paraspinal muscularity were associated with less favourable postoperative pain or disability outcomes in several studies, although findings varied across measures and follow‐up periods. Associations were reported for both postoperative scores and improvement from baseline, which represent distinct outcomes. While FI was more consistently associated with outcomes (J. Chen et al. 2022; Schönnagel et al. 2024; You et al. 2023), the available findings do not reliably establish whether FI provides greater prognostic value than CSA or whether either measure consistently predicts recovery across surgical approaches.

### 3.4 Change in Paraspinal Cross Sectional Area Correlates With Patient‐Reported Outcome Measures

Associations between postoperative muscle measures and PROMs varied by outcome and surgical subgroup. Two studies examined associations between pre‐ to postoperative changes in MF CSA and postoperative PROMs (Table 3). Both found a direct correlation in at least one study arm, but the strength of correlation was inconsistent. Chen et al. (2021) reported an association between MF CSA change and ODI at 12 months in patients undergoing three‐segment TLIF (*r* = 0.36; *p* < 0.03), but not in those undergoing two‐segment TLIF (*r* = 0.11; *p* = 0.52) (K. Chen et al. 2021). While patients had a significant reduction in VAS score at 12 months, associations with VAS were not statistically significant in either subgroup. Fan et al. (2010) reported associations between MF CSA change and both ODI (*r* = −0.718; *p* < 0.001) and VAS (*r* = −0.798; *p* < 0.001) (Fan et al. 2010). The authors also reported a significant difference between approaches, with VAS and ODI scores significantly lower for minimally invasive PLIF (VAS = 1.7 ± 0.6, ODI = 9.7 ± 3.2) compared to the conventional approach (VAS = 3.5 ± 0.6, ODI = 23.1 ± 6.4) at final follow‐up.

Overall, both studies reported associations between MF CSA change and ODI in at least one surgical subgroup, whereas an association with VAS was reported only by Fan et al. (2010).

## 4 Discussion

### 4.1 Postoperative Outcomes Do Not Consistently Scale With Preoperative Changes in Multifidus and Erector Spinae Cross‐Sectional Area and Fatty Infiltration, Before and After Lumbar Interbody Fusion

Across the included studies, MIS procedures were generally associated with a smaller decline in postoperative paraspinal muscle size and quality compared with COS, although this pattern was not uniform and varied by surgical approach (Table 1). Lower preoperative FI, and less consistently, larger preoperative CSA, was associated with greater improvement in PROMs in most, but not all, prognostic studies. While previous studies have identified paraspinal muscle quality as an important predictor of surgical outcomes following LIF, to our knowledge, this systematic review is the first to integrate both pre‐ and postoperative imaging biomarkers with PROMs while directly comparing LIF techniques, synthesising current literature for LIF techniques, and illustrating areas of unexplored research in muscle quality biomarkers and functional outcomes (Gengyu et al. 2022; Han et al. 2023).

Although improvements in PROMs are expected following successful LIF, the extent of paraspinal muscle damage measured by established biomarkers does not consistently correlate with the degree of postoperative functional recovery. The inconsistency between muscle damage biomarkers over various MIS and COS LIF methods and the increase in PROMs suggests that additional factors may influence functional recovery. Consequently, this indicates that current measures might not sufficiently capture subtle or functionally relevant muscle injuries and highlights the necessity for further research to elucidate the precise mechanisms of paraspinal muscle injury and recovery following LIF.

### 4.2 Preoperative Fatty Infiltration Was More Consistently Associated With Postoperative PROMs Than Cross‐Sectional Area

Analysis of the current literature revealed an association between the pre‐operative paraspinal muscle quality and size, and postoperative ODI and VAS scores. This result is consistent with past literature which has reported associations between paraspinal morphometry and pain scores, PROMs, and postsurgical complication rates for spine surgery more generally (Gengyu et al. 2022). In the majority of studies included in this review, greater pre‐ to postoperative CSA reduction and increased FI were both associated with worse improvement in ODI and VAS. While both CSA and FI were predictors, the association of FI and outcomes was supported by more studies and more strongly supported by studies which used both metrics. The seeming superiority of FI as an indicator of muscle quality is also supported by studies on other muscle groups, which show that losses in strength and mass are often not directly proportional (Delmonico et al. 2009). FI also impairs mitochondrial function and promotes chronic inflammation, both of which negatively impact muscle contractility and regenerative capacity (Zhu et al. 2024). In this way, FI can provide more insight into muscle dysfunction than CSA, which can be falsely elevated by pathology such as FI or oedema. This pattern has also been observed in the lumbar paraspinal muscles themselves. In a cross‐sectional study of patients with mechanical low back pain, lumbar disc herniation, and healthy controls, the functionality of the paraspinal muscles was measured using trunk endurance times, including the Biering‐Sørensen test, and was correlated with MRI‐measured paraspinal FI and CSA. They concluded that the test times shortened as the amount of FI of the muscles increased, whereas endurance was not significantly associated with CSA (Yazici and Yerlikaya 2022). Although that population was non‐surgical, these findings suggest that FI captures clinically measurable paraspinal function, which may help explain why FI was more consistently associated with postoperative PROMs than CSA in the studies reviewed here.

### 4.3 Minimally Invasive Techniques Are Associated With Less Postoperative Change in Paraspinal Muscle Quality and Size

Current literature states that improvements in PROMs are greater for LIF patients treated with an MIS approach compared with a COS approach (Ali et al. 2024). Our results were broadly consistent with the current literature, but the degree of muscle preservation depended on the surgical approach (Table 1). Of the studies included in our review evaluating COS approaches, seven of nine found a significant decrease in paraspinal muscle quality and size, as measured by muscle CSA and FI. For MIS, two of twelve studies reported significant decreases in paraspinal muscle quality and size, indicating that atrophy and FI could be due to approach disrupting muscle, rather than effects from the long‐term presence of instrumentation or the immobilisation of the spinal segment. In a head‐to‐head comparison of MIS and COS techniques, four of six studies found smaller declines after MIS in at least one muscle or setting (Fan et al. 2010; Fu et al. 2020; K. Chen et al. 2021; Putzier et al. 2016), and two found no difference (Kameyama et al. 2023; Min et al. 2009). This advantage was often selective: it applied to the multifidus but not the longissimus in the only randomized trial (Putzier et al. 2016), to the erector spinae but not the multifidus in another study (Fu et al. 2020), and to three‐level but not two‐level TLIF (K. Chen et al. 2021).

MIS interventions are associated with faster postoperative recovery, LBP scores, and improved functional mobility compared with COS procedures, although this interpretation rests on indirect comparisons between studies. Compared to COS, MIS interventions utilise a smaller incision, and require less dissection and retraction, reducing the potential for direct injury to muscles, nerves, and supportive connective tissue. In addition, MIS techniques may cause less blood loss and require shorter operation durations, leading to decreased likelihood of ischaemic injury (Pennington et al. 2018). Surgical retraction is another hypothesised mechanism for intraoperative paraspinal damage, whereby pressure on the muscles while retracted for extended periods leads to decreased perfusion and ischaemic injury (Stevens et al. 2006). Since COS methods involve wider dissections and more retraction than MIS approaches, this mechanism would also be consistent with the differences in muscle damage seen.

### 4.4 Postoperative Decline in Multifidus CSA Was Associated With Worse Disability

It appears likely from the results of Chen et al. (2021) and Fan et al. (2010) that greater atrophy was associated with worse ODI, although these designs cannot show whether atrophy contributes to disability or reflects more extensive surgery. It is understandable that the effect of intraoperative muscle damage is more supported by ODI than by other outcome measures, as muscle strength and quality are directly necessary for many of the functional abilities measured by ODI. For Chen et al. (2021), the absence of a significant correlation between CSA and ODI for two‐segment TLIF may be due to the lack of significant muscle damage seen for both conventional and minimally invasive two‐segment TLIF, where the effects of muscle atrophy on clinical outcomes would be too small to be observed with limited sample size (*n* = 81) (K. Chen et al. 2021).

This effect could also be indirectly caused by a decrease in stability of the vertebral segments with decreased muscle mass, or that decreased flexibility of the fused level plays a part in muscle atrophy. To further investigate this relationship, Strube et al. (2016) observed the effects of motion restriction on the paraspinal muscles following LIF by comparing anterior LIF, which does not surgically disrupt paraspinals, to total disk replacement, which maintains mobility of the spine (Strube et al. 2016). They found minimal differences in paraspinal muscle volume and FI, suggesting that motion restriction alone may not explain the changes seen after posterior approaches.

### 4.5 Limitations and Future Work

The findings of this review emphasise the need to prioritise muscle preservation during LIF to optimise patient outcomes. The evidence supporting the benefits of MIS approaches highlights their potential as the preferred surgical technique in terms of muscle damage only. However, this evidence alone is insufficient to recommend a preferred approach overall, which also depends on pathology, number of levels, and surgeon experience. Additionally, the identification of FI and CSA as predictors of postoperative outcomes suggests that preoperative assessments of muscle quality and size merit prospective evaluation as part of surgical planning. These measures may help physicians better counsel patients on expected recovery and prognosis, and identify patients who can potentially benefit from targeted muscle‐preserving surgical techniques and prehabilitation. These assessments could also help stratify patients by risk and identify those at greater likelihood for poorer postoperative recovery, particularly those with lower CSA or higher FI, and may guide surgical planning accordingly. Although none of the included studies evaluated a rehabilitation intervention, better PROMs post LIF should be a priority for the field, and a testable implementation of pre‐rehabilitation trials, where patients perform exercises for key muscle groups to improve outcomes and shorten recovery times, should be considered as a priority for future research. These programs have been found to be effective for spinal stenosis patients in initial studies, but larger trials are needed to validate their benefits (Marchand et al. 2021). These findings could also support postoperative rehabilitation to recover muscle mass or quality lost due to the surgical intervention, specifically through targeted interventions aimed at improving paraspinal muscle quality and size using imaging endpoints (FI and CSA) and PROMs rather than generalised rehabilitation programme alone, though it is unknown to what extent this is possible.

Limitations to this systematic review include variability in study design, differences in imaging modalities used to assess muscle quality, lack of standardisation in quality‐of‐life measures, and potential biases inherent to retrospective analyses. We consider the certainty of evidence moderate: we included one study that was randomized, most were small observational studies, three out of twenty were at serious risk of bias, and studies differed in imaging modality, muscle metrics, and follow‐up duration. Further research with standardized methodologies and advanced imaging techniques is needed to clarify the relationship between intraoperative muscle damage and postoperative outcomes. Cho et al. (2020) even demonstrated that in a single patient population, there was a difference in findings depending on whether muscle atrophy was measured by CT or MRI using manual segmentation. As multiple studies suggest FI may be a superior measure of muscle quality, we expect that further studies using different radiology and biomarker imaging modalities may reveal even more about the pathology underlying the proposed damage mechanisms. Future studies should also pair imaging with clinical measures of paraspinal function. Simple endurance tests such as the Biering‐Sørensen test track paraspinal FI in patients with low back disorders (Yazici and Yerlikaya 2022), and near infrared spectroscopy and electromyography offer additional functional metrics (Demoulin et al. 2007). Measuring these before and after LIF could help determine whether imaging changes translate into functional deficits and could provide low‐cost endpoints for the rehabilitation trials proposed above. Ultimately, evaluating a single patient population with a variety of modalities is likely necessary to comprehensively understand the type of muscle damage mechanism occurring during LIF. Understanding the mechanism of muscle injury, and muscle healing would provide valuable insight into ways to mitigate intraoperative muscle injury in the development of new MIS techniques. Future research should prioritise developing rehabilitation programs targeting these muscle groups to enhance post‐operative recovery, and the programs would also benefit from a better‐informed model of damage.

## 5 Conclusion

This systematic review demonstrates that there is a detectable postoperative decrease in muscle CSA and increase in FI following LIF intervention, although these changes vary across muscles and surgical approaches. Most, but not all, comparative studies suggested better preservation of paraspinal muscle morphology with MIS than with COS. Preoperative muscle quality increases, particularly lower FI and smaller pre‐ to postoperative decreases in muscle size correlated with better PROMs in several studies, but these relationships were inconsistent across outcomes and procedures. Heterogeneity and the predominantly observational evidence limit conclusions regarding causality and the magnitude of differences between approaches. Development of rehabilitation protocols targeting paraspinal muscles may offer a promising avenue for improving surgical outcomes. However, the mechanisms and morphological changes underlying these relationships are not yet clear, highlighting the need for further investigation, including studies using advanced imaging modalities to better understand muscle injury and recovery processes.

## Author Contributions

T.T., N.K., C.C., and R.S. contributed to the extraction of the data and preparation of the initial draft. L.H., and Y.‐K.J. contributed to the conceptualisation of the study aims and revision of the manuscript. Y.‐K.J. supervised the study.

## Ethics Statement

This study is a systematic review of previously published studies and did not involve the collection of new data from human participants. IRB approval and informed consent were therefore not required.

## Conflicts of Interest

The authors declare no conflicts of interest.

## Supporting information


**Table S1:** Full electronic search strategy for each database.

## Data Availability

Data sharing not applicable to this article as no datasets were generated or analysed during the current study.
