# Supplementary material for: Paraspinal Muscle Quality and Functional Outcomes Following Lumbar Fusion: A Systematic Review
Source: Musculoskeletal Care. 2026 Nov 7;24(4):e70299. doi: 10.1002/msc.70299 (PMC13744417; doi:10.1002/msc.70299)
Supplement: Supplementary file 1 [file MSC-24-e70299-s001.docx]

**Table S1. Full electronic search strategy for each database.**

Three databases were searched from database inception to February 27th, 2025 with no date or language restrictions. In each database, two concept blocks (muscle groups and surgical techniques) were combined with the Boolean operator AND, and synonyms within each block were combined with OR. Terms were applied to an all-field option including the title, abstract, and keyword fields. No additional filters were used. Non-English records were excluded during screening.

| **Database** | **Search string** | **Records (n)** |
| --- | --- | --- |
| **PubMed (MEDLINE)** | ("back muscle" OR "paraspinal muscle" OR "multifidus" OR "paravertebral" OR "erector spinae") AND ("lumbar fusion" OR "lumbar interbody fusion" OR "TLIF" OR "PLIF" OR "LLIF" OR "ALIF") | 231 |
| **Scopus** | ("back muscle" OR "paraspinal muscle" OR "multifidus" OR "paravertebral" OR "erector spinae") AND ("Lumbar fusion" OR "lumbar interbody fusion" OR "TLIF" OR "PLIF" OR "LLIF" OR "ALIF") | 386 |
| **Web of Science** | TS=("back muscle" OR "paraspinal muscle" OR "multifidus" OR "paravertebral" OR "erector spinae") AND TS=("lumbar fusion" OR "lumbar interbody fusion" OR "TLIF" OR "PLIF" OR "LLIF" OR "ALIF") | 365 |
| **Total records identified** | | **982** |

*Note: Record counts reflect results retrieved on the date of search, prior to duplicate removal.*
